# NPFFR2 Contributes to the Malignancy of Hepatocellular Carcinoma Development by Activating RhoA/YAP Signaling

**DOI:** 10.3390/cancers14235850

**Published:** 2022-11-27

**Authors:** Yuna Shin, Wonhee Jung, Mi-Yeon Kim, Dongjo Shin, Geun Hee Kim, Chun Ho Kim, Sun-Hoo Park, Eung-Ho Cho, Dong Wook Choi, Chul Ju Han, Kee Ho Lee, Sang-Bum Kim, Hyun Jin Shin

**Affiliations:** 1 Team of Radiation Convergence Research, Korea Institute of Radiological & Medical Sciences, Seoul 01812, Republic of Korea; 2 Radiological and Medico-Oncological Sciences, University of Science and Technology, Daejeon 34113, Republic of Korea; 3 Division of Radiation Biomedical, Research Korea Institute of Radiological and Medical Sciences, Seoul 1812, Republic of Korea; 4 Department of Pathology, Korea Institute of Radiological and Medical Sciences, Seoul 01812, Republic of Korea; 5 Department of Surgery, Korea Institute of Radiological and Medical Sciences, Seoul 01812, Republic of Korea; 6 Department of Internal Medicine, Korea Institute of Radiological and Medical Sciences, Seoul 01812, Republic of Korea

**Keywords:** NPFFR2, GPCR, NPFF, RhoA, YAP, hepatocellular carcinoma, invasion, migration

## Abstract

**Simple Summary:**

G protein–coupled receptors (GPCRs) are the most critical protein group for drug development, targeting about 35% of approved drugs. As their abnormal activation causes many diseases, including cancer, it is beneficial to discover novel GPCRs that are aberrantly expressed in cancer and function in cancer progression. We discovered that neuropeptide FF receptor 2 (NPFFR2) is aberrantly expressed in liver cancer and promotes malignancy by enhancing the activity of RhoA/YAP, and inhibiting NPFFR2 dramatically reduced the malignant phenotypes. We expect that these findings provide a novel potential target for cancer treatment.

**Abstract:**

G protein–coupled receptors (GPCRs) are a diverse family of cell surface receptors implicated in various physiological functions, making them common targets for approved drugs. Many GPCRs are abnormally activated in cancers and have emerged as therapeutic targets for cancer. Neuropeptide FF receptor 2 (NPFFR2) is a GPCR that helps regulate pain and modulates the opioid system; however, its function remains unknown in cancers. Here, we found that NPFFR2 is significantly up-regulated in liver cancer and its expression is related to poor prognosis. Silencing of NPFFR2 reduced the malignancy of liver cancer cells by decreasing cell survival, invasion, and migration, while its overexpression increased invasion, migration, and anchorage-independent cell growth. Moreover, we found that the malignant function of NPFFR2 depends on RhoA and YAP signaling. Inhibition of Rho kinase activity completely restored the phenotypes induced by NPFFR2, and RhoA/F-Actin/YAP signaling was controlled by NPFFR2. These findings demonstrate that NPFFR2 may be a potential target for the treatment of hepatocellular carcinoma.

## 1. Introduction

G protein–coupled receptors (GPCRs) are a major drug target, as this protein group is the largest family of receptors involved in a variety of physiological processes and is a critical initiator of physiological network signaling involved in various diseases [1,2]. Although GPCRs are targets for one-third of approved drugs, only approximately 100 unique GPCRs from a total of approximately 800 genes are current targets [3,4], which indicates that there are many GPCRs that may serve as potential therapeutic targets that remain to be studied. 

NPFFR2 (Neuropeptide FF receptor 2) is a member of the class A family of GPCRs, activated by the neuropeptide FF group (NPFF, NPAF) belonging to the RFamide peptide [5], and is known to function in pain modulation [5,6,7] and regulation of the opioid system [8]. NPFFR2 overexpressed transgenic mice exhibited increased activation of pain–regulated brain regions after painful stimulation and up–regulated inflammatory mediators [7]. Recently, it has been implicated in thermoregulation and stress–related depressive behavior via regulation of the hypothalamic circuitry [9,10]. Likewise, most studies on NPFFR2 have focused on the nervous system.

Neuro–inflammatory processes modulated by neuropeptide ligands are also closely related to the degree of pain experienced by cancer patients [11]. Moreover, there have been many reports demonstrating that neuropeptides and their receptors contribute to cancer aggressiveness [12,13,14]. Neuropeptides, such as bombesin, neurotensin, and neuropeptide Y are produced from tumors and bind to tumors autocrinally to stimulate growth [15,16].

Hepatocellular carcinoma (HCC) is a common cause of cancer–related mortality [17,18]. There are few effective targeted anticancer drugs for HCC [19]. Although the combination of targeted therapy with immune checkpoint inhibitors has become a popular option for advanced HCC, a large proportion of patients are unresponsive to this therapy [20,21,22]. Therefore, there is an urgent need to uncover potential therapeutic targets for advanced HCC. 

In this study, we investigated the role of NPFFR2 in HCC. Here, we showed that NPFFR2 is overexpressed in HCC and associated with a poor prognosis and identified that NPFFR2 is critical for tumor cell survival and aggressiveness, and these functions in cancer depend on RhoA and its downstream signaling YAP pathway. This study presents a novel function of the RFamide peptide receptor NPFFR2 in cancer progression and suggests a potential therapeutic target for advanced HCC. 

## 2. Materials and Methods

### 2.1. HCC Patient Tissues

Specimens were collected from patients with HCC who underwent hepatic surgery between 1992 and 2004 at the Korea Cancer Center Hospital (Seoul, Republic of Korea). In total, 108 HCC samples, including 30 pair–matched samples, were used. Among these, 20 pairs of HCC and adjacent liver tissue samples were used for RT–PCR. Liver tissues obtained during resection due to liver metastasis from other primary tumors were defined as normal liver tissue. 

### 2.2. TCGA Data Analysis

Recurrence–free survival curves of NPFFR2 were analyzed based on the Kaplan–Meier plotter using mRNA sequencing data of HCC from the Cancer Genome Atlas (TCGA) dataset (http://www.kmplot.com (accessed on 1 September 2022)) [23]. Significance was defined as a log–rank *p*-value of <0.05. The relative expression of NFFR2 in normal tissues and tumor sub–groups from TCGA dataset was further analyzed using the UALCAN platform (http://ualcan.path.uab.edu (accessed on 1 September 2022)).

### 2.3. RT–PCR and Real–Time PCR

Total RNA from frozen tissues or cancer cell lines were purified using Qiagen RNeasy Mini Kit (Qiagen, Valencia, CA, USA) [24] or RNeasy Mini Kit (GeneAll, Seoul, Republic of Korea), respectively. The frozen tissues were homogenized in RNA lysis buffer and briefly centrifuged to remove cell debris. cDNA was synthesized from the purified RNAs using iScript reverse Transcription Supermix (Bio–Rad, Hercules, CA, USA). Semi–quantitative RT-PCR was performed using the Maxine PCR PreMix Kit (iNtRON Biotechnology, Seongnam, Republic of Korea) and real–time PCR was performed using the SYBR Green method with the KAPA SYBR FAST universal qPCR kit (Kapa Biosystems, Wilmington, MA, USA), or the TaqMan method using the iQ^tm^Supermix (Bio-Rad). The forward and reverse primer and probe sequences are as follows: NPFFR2, (for RT-PCR) 5′-TTTTTGTGCATGATGGGAA/5′-TATTCCCTGGACCAATCCAC; NPFFR2 (for real–time PCR) 5′-TCGCAGCTTCAGTCTTTACG, 5′-GATGGCTAGGACCCAGAT GA, 56-FAM/AGGGTAGAC/ZEN/CAC ACACTGGAACCTAT/3IABkFQ; B2M (for real-time PCR and RT–PCR) 5′-AAGGACTGGTCTTTCTATCTCTTGTA, 5′-ACTATCTTGGGCTGTGACAAAGTC, 56-FAM/TGGTTCACA/ZEN/CGGCAGGCATAC TCA/3IABkFQ; CTGT, 5′-GAAGCTGACCTGGAAGAGAACA, 5′-CGTCGGTACATACTCCACAGAA; Cyr61, 5′-CATTCCTCTGTGTCCCCAAGAA, 5′-TACTATCCTCGTCACAGACCCA; β-actin, 5′-AGCGAGCATCCCCCAAAGTT, 5′-GGCACGAAGGCTCATCATT. The relative expression was analyzed using the comparative threshold cycle (2^−ΔΔCt^) method. Data are presented as the mean ± SD of triplicate experiments. 

### 2.4. Statistical Analysis

Statistical analyses of survival curves and box plots were performed using SPSS software version 22 (SPSS Institute, Chicago, IL, USA). Survival curves were plotted using the Kaplan–Meier method, and differences in survival rates were analyzed using a log–rank test. The criteria dividing the tumor group were the average fold change in NPFFR2 expression in total HCC tissues compared to the expression in the non–tumor tissues. The *p*-value was determined using log–rank analysis. The prognostic values of overall survival and recurrence–free survival were determined using a Cox regression model. Survival rates were calculated based on scoring, death, or recurrence as the respective events. Significance was set at *p* < 0.05. 

Statistical methods for other data were analyzed as mean ± SD. Significant differences between the two means were assessed using the Student’s *t*-test (unpaired, two–tailed). Differences were considered significant at *p* ≤ 0.05, and *p*-values of >0.05 were considered non–significant (n.s.).

### 2.5. Cell Culture and Reagents

Huh7 cells and all SNU HCC cell lines were cultured in RPMI-1640 medium (LM011-01; Welgene, Daegu, Republic of Korea), Hep3B, SK-Hep-1, and HepG2 cells were cultured in Minimum Essential Medium (LM007-7; Welgene), and all normal fibroblasts were cultured in Dulbecco’s Modified Eagle Medium (LM 001-05). The medium was supplemented with 10% (*v/v*) fetal bovine serum (FBS; 43640, JRS, CA, USA) and 1% (*w*/*v*) antibiotics (LM203-01, Welgene). The cells were cultured in a humidified CO_2_ incubator at 37 °C (5% CO_2_). 

The NPFF peptide Phe-Leu-Phe-Gln-Pro-Gln-Arg-Phe-NH2 was purchased from Tocris Bioscience (Bristol, UK) and dissolved in dH_2_O. The Rho kinase (ROCK) inhibitor, Y27632, was purchased from Sigma-Aldrich (St. Louis, MO, USA) and dissolved in DMSO.

### 2.6. siRNAs, Plasmids, and Transfection

The small interfering RNA (siRNA) duplexes were synthesized by Bioneer (Daejeon, Republic of Korea). The sequences were as follows: NPFFR2 siRNA #1, GGAAUUAGUGAUGGAAGAA, and NPFFR2 siRNA #2, GACUCUAAUGAUGCUCUCA. The negative control was purchased from Bioneer. Cells were transfected with siRNA at a concentration of 20 nM using RNAiMAX (Invitrogen, Carlsbad, CA, USA) in Opti-MEM (Invitrogen). After incubation for 12 h, the medium was changed to complete medium and further incubated for 48 h. 

Plasmids expressing NPFFR2 (pCDNA3-FLAG-NPFFR2) were kindly gifted by Frederic Simonin (French National Center for Scientific Research) [25] and the plasmid was sub–cloned into pLVX-IRES-puro. For transient overexpression, plasmids were transfected using TurboFect (Thermo Fisher Scientific Inc., Waltham, MA, USA). DNA and TurboFect were mixed in Opti-MEM (with the following ratio—DNA 1 μg: Turbofect 1.5 μL), and the prepared cells were transfected following the manufacturer’s protocol. 

### 2.7. Lentivirus Infection

pLVX-NPFFR2 or its backbone vector, pLVX-IRES-puro (4 μg) was co-transfected with lentiviral structure proteins (pCMV-dR8.2 (4 μg) and pVSV-G (2 μg)) into 293T cells plated on a 100 mm dish with 80% confluency using 12 μL TurboFect. The pLVX-IRES-Puro backbone vector was used as the negative control. After 24–30 h, the medium was collected and filtered. Cells were incubated in media containing the lentivirus and 8 μg/mL polybrene for 6 h. After 2–3 days, sub–cultured cells were treated with puromycin (0.5 μg/mL) and selected resistant cells were used within five passages. 

### 2.8. Invasion Assay

Transwells (3422, Corning, NY, USA) were coated with 20 μL Matrigel (1 mg/mL, Corning) and allowed to dry thoroughly on a clean bench. Cells (2 × 10^4^) were seeded in the upper compartment of the chamber in 100 μL serum–free medium. Complete medium containing 10% FBS was placed in the bottom compartment of the chamber. For peptide treatment, NPFF was added to both the upper and lower chambers at a concentration of 3 μM. After 30–36 h of incubation, the cells were fixed and stained with Hemacolor Solution (Merck, Darmstadt, Germany). In a single experiment, stained cells were counted based on photographs acquired using a light microscope at 40× magnification and the average cell number was calculated based on three technical replicates. Graphs were plotted based on relative cell numbers to the control and generated using mean values from three independent experiments. 

### 2.9. Migration Assay

For the migration assay, a wound healing assay was performed using a SPLScar Block (201935, SPL Life Sciences, Pocheon, Republic of Korea). Cells (1 × 10^4^) were seeded on SPLScar Block and confluent cells were starved for 6 h in an FBS–free cell culture medium. A cell–free gap was generated by detaching the silicon block, and the medium was replaced with a complete medium containing inhibitors or peptides according to experimental conditions. Cell images were acquired every 12 h using a light microscope until the cells filled the gaps. The width of the cell–free gap was measured in triplicate. Relative migration activity was determined as followed: Ratio of migration = [(gap width at 0 h) − (gap width at measure time)]/(width at 0 h). 

### 2.10. Colony Formation and Soft Agar Assays

For the colony formation assay, siRNA–transfected cells were used. After 36 h of transfection, 3000 cells were seeded on 60 mm plates and incubated for approximately 10 days until colonies were formed. Colonies were fixed with 5% formaldehyde and visualized using crystal violet (0.5% crystal violet prepared in 25% methanol).

For the soft agar assay, Huh7 stable cell line overexpressing NPFFR2 was established by infecting with lentivirus and used under five passages. A bottom layer of 1% agar in culture media was poured into 12-well plates and a top layer of 0.7% agar in culture media containing 1500 cells was poured on the bottom layer. Several drops of the culture medium were added every 3–4 days. After three weeks, the agar was stained with 0.05% crystal violet.

### 2.11. Western Blotting and RhoA Assay

Harvested cells were purified using TNN buffer (50 mM Tris-Cl (pH 7.4), 150 mM NaCl, 0.5% NP-40, and 1 mM EDTA) supplemented with protease inhibitor cocktail (GenDEPOT, Barker, TX, USA). Protein–transferred membranes were blocked using the TBS-T buffer containing 5% skim milk and incubated with the primary antibodies diluted in TBS-T buffer containing 5% BSA. After washing three times with TBS-T, the membranes were incubated with horseradish peroxidase–conjugated secondary antibodies (A120-101P and A90-116P, Bethyl Laboratories, Montgomery, TX, USA) in TBS-T with 5% skim milk. The membranes were then reacted with Western blotting luminol reagent (sc-2048; Santa Cruz Biotechnology, Dallas, TX, USA). The following primary antibodies were used: p-ERK (Thr202/Tyr204, #9101, Cell Signaling Technology, Danvers, MA, USA), ERK (#9102, Cell Signaling Technology), p-AKT (Ser473, #4060, Cell Signaling Technology), p-PKCαI/βII (Thr638/641, #9375, Cell Signaling Technology), and β-actin (sc-47778, Santa Cruz Biotechnology). In the experiment with peptide, cells were starved in serum-free medium for 12 h, and then NPFF was added directly to the incubating media.

RhoA activity was assessed in Huh7 cells transfected with NPFFR2 using a RhoA Activation Assay Kit (BK036; Cytoskeleton, Inc., Denver, CO, USA), according to the manufacturer’s protocol. Protein (1 mg) diluted in 0.5 mL lysis buffer was mixed with 50 μg Rhotekin RBD Agarose beads and incubated on a rotator at 4 °C for 1 h. The washed beads and cell lysates were subjected to Western blotting, and RhoA was detected. 

### 2.12. Immunofluorescence

For phalloidin and YAP staining, plasmids or siRNAs was transfected into Huh7 cells or SNU475 cells, respectively, and cells were grown on coverslips. After 48 h of transfection, cells were fixed with 5% formaldehyde prepared in PBS, and non–specific antigens were blocked with 2% (*w*/*v*) skim milk in PBS containing 0.1% Triton X-100 for 1 h at room temperature. Cells were probed with phalloidin (A12380, Invitrogen) or incubated with primary antibodies against YAP (sc-376830, Santa Cruz Biotechnology), prepared in blocking buffer for 2 h. Antibody–stained sections were incubated with a fluorescent–conjugated secondary antibody (Thermo Fisher Scientific) for 1 h at room temperature. The nuclei were then stained with Hoechst 33342 and the cells were mounted using mounting solution (90% glycerol, 10 mM Tri-Cl, pH 8.8). For staining NPFFR2, primary antibody targeting NPFFR2 (NPB300-169, Novus Biologicals, Littleton, CO, USA) were used. Images were obtained using a confocal microscope LSM510 (Carl Zeiss, Jena, Germany).

## 3. Results

### 3.1. NPFFR2 Is Overexpressed in HCC Tissues and the Expression Is Related to Poor Prognosis

**Figure 1:**
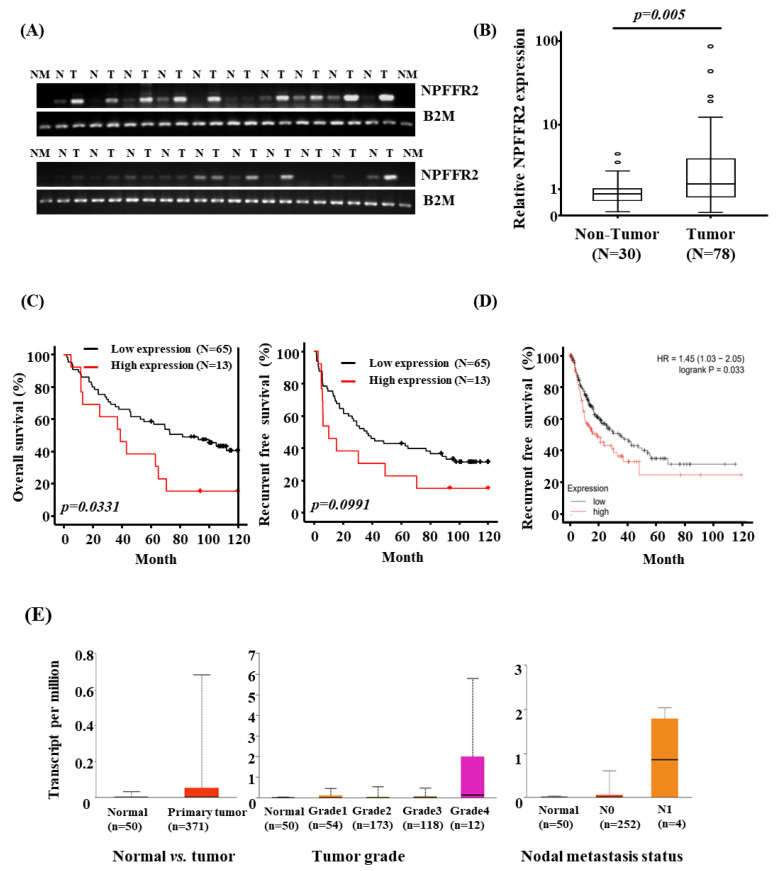
Analysis of NPFFR2 expression in HCC tissues. (**A**) NPFFR2 mRNA expression was compared in HCC (T, tumor) and adjacent liver tissues (N, non–tumor) using semi–quantitative RT–PCR. (**B**) Boxplot analysis depicting increased NPFFR2 expression in HCC tissues (n = 78) compared with adjacent liver tissues (n = 28). Values deviating from the quartile groups were individually marked with dots. (**C**) Kaplan–Meier survival analysis for NPFFR2 expression in (78) HCC patients (n = 78, low expression, black line < 4.43–fold and high expression, red line ≥ 4.43–fold). (**D**) Comparison of expressions by recurrence–free survival analysis. Recurrence–free survival curves of NPFFR2 were analyzed from the Kaplan–Meier plotter using mRNA sequencing data of HCC (n = 312) in TCGA dataset. (**E**) The relative expression of NPFFR2 in normal tissues and tumor subgroups was analyzed from TCGA dataset using the UALCAN platform. NPFFR2 expression was compared in normal and cancerous tissues (**left**), across tumor grades (**middle**), and between the presence or absence of nodal metastasis (**right**) from TCGA data using the UALCAN platform.

To validate the expression of NPFFR2 in cancer, we analyzed its expression in HCC patient tissues and various cancer cell lines. RT–PCR was performed using 20 pairs of HCC and adjacent non–tumorous liver tissues (Figure 1A). The majority of the HCC tissues (75%) exhibited increased expression of NPFFR2 compared with its adjacent tissue. To quantitatively validate the increased expression of NPFFR2 in HCC tissues, its expression was analyzed by real–time PCR using 30 samples of non–tumorous liver tissues and 78 samples of HCC tissues (Figure 1B). As a result of the analysis, a box plot confirmed that the expression of NPFFR2 increased in HCC tissues compared with the surrounding liver tissue. 

The results of expression data in HCC tissues and patients’ information were used to evaluate whether NPFFR2 could be a prognostic factor. Kaplan–Meier survival curves indicated that overall survival (*p* = 0.033) and relapse–free survival (*p* = 0.099) were poor in patients with high NPFFR2 expression (Figure 1C). Survival curves were also analyzed for 364 patients with HCC in TCGA public database. Poor relapse–free survival was related to poor prognosis with five statistically significant cutoff values (*p* = 0.033) (Figure 1D). The increased expression of NPFFR2 in HCC was also confirmed in TCGA database, and interestingly, the expression was notably increased in higher grade and nodal metastasis status (Figure 1E). 

### 3.2. The Expression Levels of NPFFR2 Is Closely Associated with the Survival and Proliferation of HCC Cells

**Figure 2:**
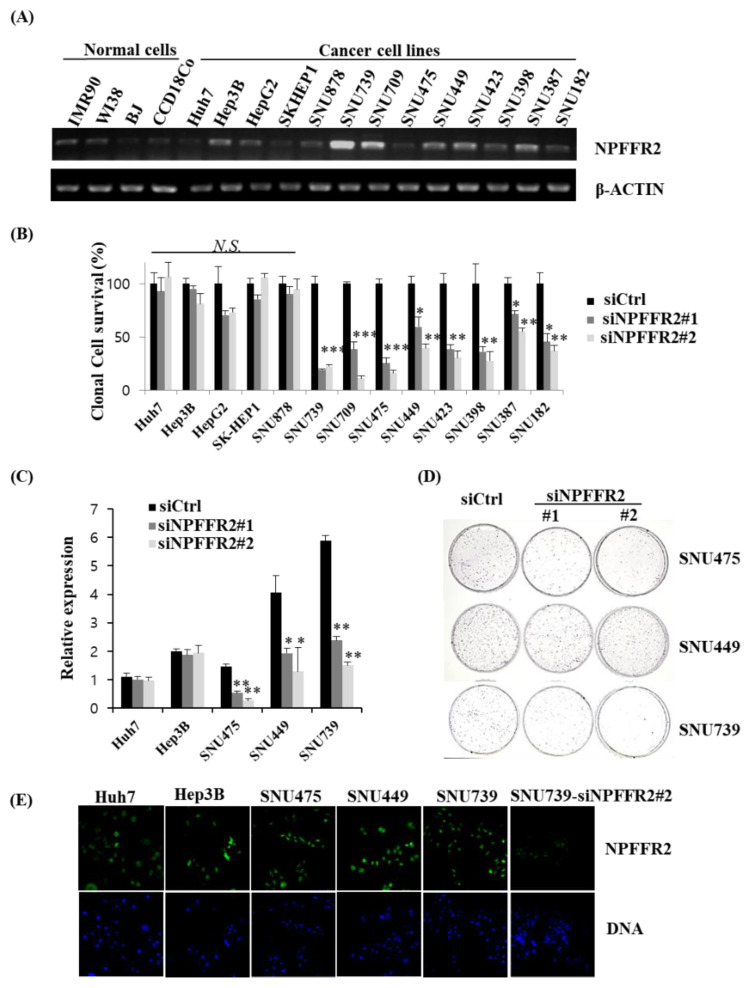
NPFFR2 is essential for cancer cell survival. (**A**) mRNA expression of NPFFR2 in normal cells and various HCC cell lines was analyzed by RT–PCR. (**B**,**D**) To evaluate the function of NPFFR2 on cancer cell survival, clonogenic survival of various HCC cell lines where NPFFR2 was depleted by siRNAs was assessed. The average percentage of the number of colonies relative to control was calculated and shown in a bar graph. This experiment was performed independently at least thrice. (**C**) The depletion of NPFFR2 was confirmed by quantifying NPFFR2 mRNA with real–time PCR in indicated cell lines. NPFFR2 expression was calculated from the mean CT value of technical triplicates and normalized with B2M. * indicates *p ≤* 0.05, ** indicates *p <* 0.01, *** indicates *p <* 0.001, *N.S.* indicates non–significant (*p* > 0.05). (**D**) Representative result of clonogenic assay performed using three cell lines in which the expression of NPFFR2 was efficiently suppressed by siRNA. (**E**) The protein expression of NPFFR2 in five HCC cell lines was assessed by immunostaining and the protein reduction in siRNA–transfected cells was also confirmed by immunostaining.

The expression of NPFFR2 was also confirmed using HCC cell lines and other cancer cell lines from various origins. We compared RNA expression in cancer cells and normal fibroblasts. The expression was relatively increased in some cancer cells, especially SNU739, SNU709, U2OS, H460, and Calu-1 (Figure 2A, Supplementary Figure S1).

To validate the function of NPFFR2 in cancer cell survival, a clonogenic assay was performed using siRNAs targeting NPFFR2. The survival of many HCC cells was reduced by NPFFR2 depletion (Figure 2B,D). The degree of inhibition of cell survival by NPFFR2 depletion correlated with some extent with the endogenous expression levels of NPFFR2 in various HCC cells (Figure 2A,B). We selected several cell lines whose viability was or was not reduced by si–NPFFR2 transfection and analyzed the knockdown efficiency of siRNAs. siRNAs worked effectively in SNU449, SNU475, and SNU739 cells, where cell survival was reduced by siRNA, whereas Hep3B and Huh7 cells, whose survival was not reduced by siRNA, exhibited no reduction in expression by siRNA (Figure 2C). Cytotoxicity is induced only when the expression of NPFFR2 is effectively suppressed implies that siRNA–mediated cytotoxicity is not an off–target effect and targeting NPFFR2 can selectively induce cytotoxicity in NPFFR2–overexpressing cancer cells. The expression of NPFFR2 in HCC cells and depletion of NPFFR2 by siRNA was also confirmed at the protein level by immunostaining (Figure 2E). The NPFFR2 protein level in five cells correlated with each mRNA level to some degree, as shown by the lower expression in Huh7 cells. In addition, it was confirmed that siRNA targeting NPFFR2 decreases the expression of NPFFR2 protein.

### 3.3. NPFFR2 Increases Invasiveness and Migration in HCC Cancer Cells

**Figure 3:**
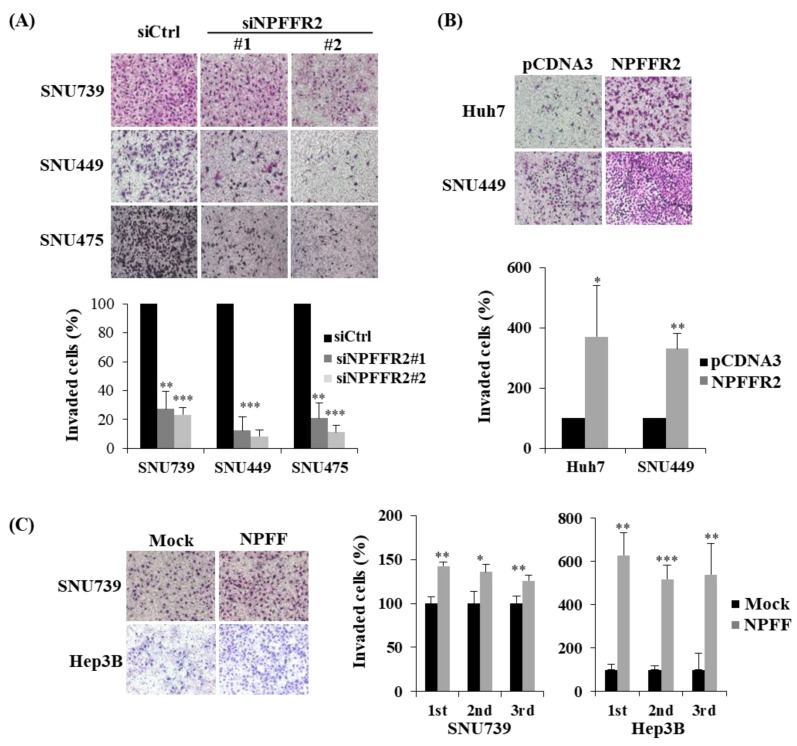
NPFFR2 increases invasiveness of cancer cells. (**A**–**C**) Transwell Matrigel invasion assay was performed with indicated HCC cells under conditions where NPFFR2 was depleted by siRNA (**A**), overexpressed by transient transfection (**B**), or activated by ligand (**C**). To verify invasion effect by regulation of NPFFR2 expression, cells transfected with siRNAs (**A**) or plasmid vector (**B**) for 24 h were seeded in a matrigel–coated chamber and incubated for 30–36 h. To verify the invasion effect of NFPPR2 ligand (**C**), cells were seeded under media conditions containing 3 μM of NPFF in both the upper and lower chamber and incubated for 30–36 h. Stained invaded cells were photographed (magnification, ×40). All data were obtained from three independent experiments and each experiment was performed in triplicates. * indicates *p <* 0.05, ** indicates *p <* 0.01, *** indicates *p <* 0.001.

As NPFFR2 is especially increased in advanced HCC with nodal metastasis according to information obtained from TCGA database, we performed an invasion assay to determine whether increased NPFFR2 expression in advanced tumors supports malignant features. siRNA–driven ablation of NPFFR2 dramatically reduced invasiveness by more than 70%–90% in the SNU739, SNU449, and SNU475 cells (Figure 3A) and transient overexpression increased invasion from three–fold to six–fold in Huh7 and SNU449 cells (Figure 3B). The invasiveness of NPFFR2 was also confirmed in NPFFR2 overexpressing stable cell lines constructed by infecting the lentivirus in Huh7, SNU475, and SNU739 cells (Supplementary Figure S2). We performed an invasion assay under conditions of NPFFR2 activation using the NPFF peptide in the SNU739 and Hep3B cells and observed that the NPFF peptide increased invasiveness in these cells (Figure 3C).

### 3.4. NPFFR2 Increases Migration in HCC Cancer Cells

**Figure 4:**
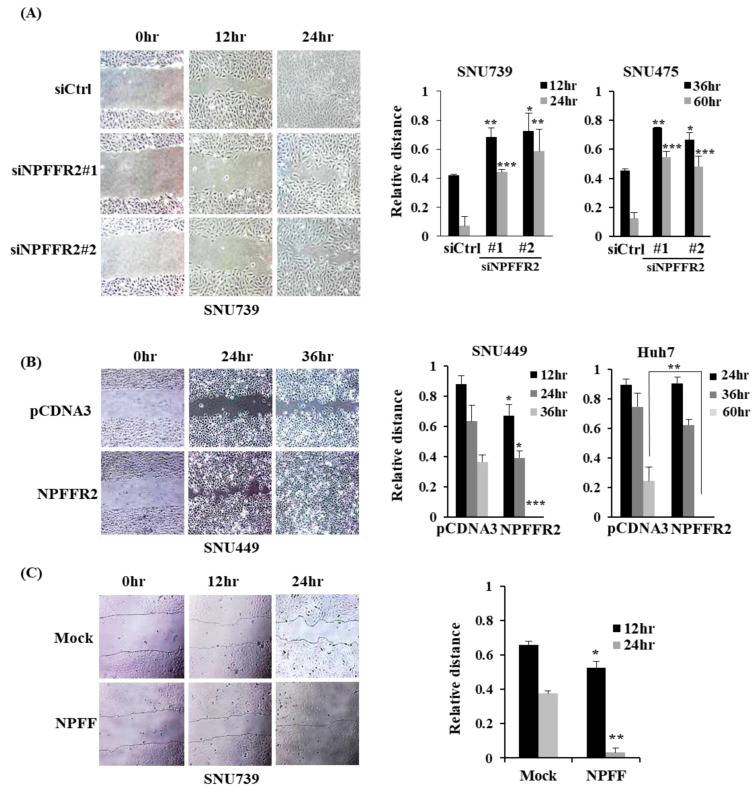
NPFFR2 increases cancer cell migration. (**A**–**C**) A wound healing assay was performed with indicated HCC cells under conditions where NPFFR2 was depleted by siRNA (**A**), overexpressed by transient transfection (**B**), or activated by ligand (**C**). To verify the migration effect by regulation of NPFFR2 expression, cells transfected with siRNAs (**A**) or plasmids (**B**) for 24 h were prepared for the assay. To verify the migration effect of NFPPR2 ligand (**C**), starved cells were stimulated with complete media containing 3 μM of NPFF. At least three independent experiments were performed, and data are presented as the average of triplicate in one representative experiment. * indicates *p* < 0.05, ** indicates *p* < 0.01, *** indicates *p* < 0.001.

We further validated the effect of migration, another integral component of metastasis, using NPFFR2. The wound closure rate decreased by NPFFR2 depletion in SNU739 and SNU475 cells (Figure 4A) and increased by NPFFR2 overexpression in the SNU449 and Huh7 cells (Figure 4B). NPFFR2 activation by the NPFF peptide also increased cell migration in SNU739 cells (Figure 4C).

### 3.5. NPFFR2 Increases Invasiveness in Cancer Cells through RhoA Signaling

**Figure 5:**
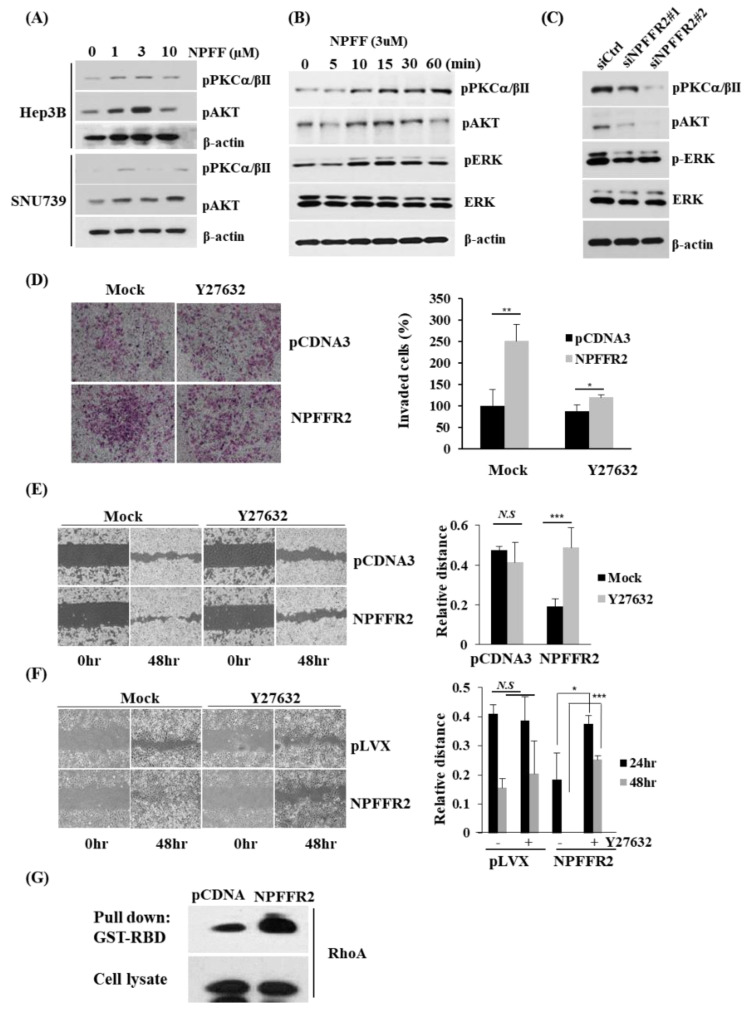
Invasiveness of NPFFR2 depends on RhoA activity. (**A**,**B**) NPFF activates GPCRs, thereby activating AKT, PKC, and ERK signaling. SNU739 (**A**,**B**) and Hep3B (**A**) cells were starved for 12 h, treated with peptides by concentration for 15 min (**A**) or by time at a concentration of 3 μM (**B**), and harvested for Western blotting. (**C**) NPFFR2 was depleted in SNU739 cells using siRNA, and cells were harvested for Western blotting after 48 h. (**D**) The effect of Rho activity on NPFFR2–induced cell invasion was analyzed4 h after transfection, Huh7 cells were seeded into a transwell, and the upper and lower wells were simultaneously treated with Y27632 (5 µM). (**E**) The effect of Rho activity on NPFFR–induced cell migration was analyzed. Transfected Huh7 cells (**E**) or SNU475 stable cell lines (**F**) were seeded in the migration chamber and confluent cells were starved for 6 h. Media was changed to complete media containing Y27632 after a generation of cell–free gap. (**D**–**F**) Experiments were performed in triplicate and the data are presented as mean ± SD. (**G**) NPFFR2 overexpression increases RhoA activity. RhoA activity assay was performed using NPFFR2–transfected Huh7 cells. Cell lysates were incubated with Glutathione Sepharose bead–bound GST–fusion protein of the Rho–binding domain of Rhotekin (GST–RBD) to capture GTP–bound RhoA. RhoA–GTP protein expression was detected using RhoA antibodies. * indicates *p ≤* 0.05, ** indicates *p <* 0.01, *** indicates *p <* 0.001, *N.S.* indicates non–significant (*p* > 0.05).

To elucidate the mechanism by which NPFFR2 increases cancer malignancy, we analyzed the expression of representative downstream signaling proteins activated by GPCR signaling in NPFFR2–depleted or NPFF–treated cells. NPFF treatment activated PKC, AKT, and ERK signaling in SNU739 and Hep3B cells (Figure 5A,B) and depletion of NPFFR2 by siRNA decreased the activity of these signaling proteins in SNU739 cells (Figure 5C). Even though NPFFR2 overexpression increased invasion and migration, we did not observe the activation of PKC, AKT, and ERK by NPFFR2 overexpression. This implies that another pathway is involved in the metastatic function of NPFFR2. 

NPFF binds to multiple G proteins such as Gαo, Gαs, and Gαi [26,27]. As RhoA–ROCK mediates the downstream effect of numerous GPCRs [28], we tested whether ROCK affects invasion induced by NPFFR2. A ROCKI and ROCKII inhibitor, Y27632, almost completely relieved the invasiveness induced by the overexpression of NPFFR2 under conditions that did not affect invasion in cells transfected with a control vector (Figure 5D). 

The effect of ROCK inhibition on the regulation of migration by NPFFR2 was also tested. Both transient NPFFR2 overexpression by transfection in Huh7 cells (Figure 5E) and stable NPFFR2 overexpression by lentiviral infection in SNU475 cells (Figure 5F) increased cell migration. Y27632 also relieved the increased cell migration by NPFFR2 overexpression under conditions that did not affect migration in cells where NPFFR2 was not overexpressed. 

Next, we verified whether RhoA was activated by NPFFR2 by measuring Rho–GTP using the RhoA Activity Assay Kit (Figure 5G). Transient NPFFR2 overexpression in Huh7 cells increased Rho activation compared to the control. Together, we concluded that the control of migration and invasion by NPFFR2 is due to RhoA activation.

### 3.6. Regulation of RhoA by NPFFR2 Affects the Formation of F–Actin and the Activity of YAP

**Figure 6:**
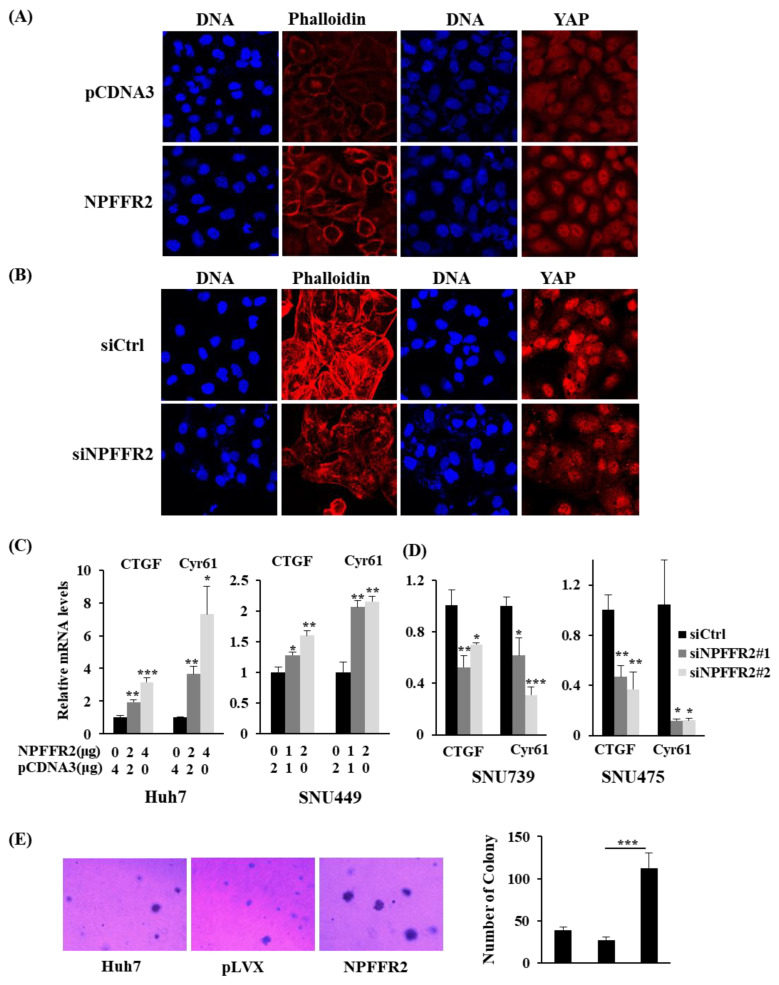
NPFFR2 increases F-actin formation to regulate YAP activation. (**A**,**B**) Representative confocal image of F-actin and YAP in Huh7 cells overexpressing NPFFR2 (**A**), in SNU475 cells where NPFFR2 is depleted (**B**). F–actin organization was visualized by staining with phalloidin and the control of YAP distribution by NPFFR2 was observed by staining using YAP antibodies. (**C**,**D**) The control of YAP activity by NPFFR2 was confirmed by analyzing the mRNA expression of *CTGF* and *Cyr61* with real–time PCR. NPFFR2 was overexpressed in Huh7 and SNU449 cells (**C**) and depleted in SNU739 and SNU475 cells (**D**). (**E**) The effect of NPFFR2 overexpression on anchorage-independent cell growth was assessed by soft agar assay using stable cell lines and their parental cells (Huh7). (**C**–**E**) At least three independent experiments were performed, and data are presented as the average of triplicate (**C**,**D**) or quadruplicate (**E**) in a representative experiment. * indicates *p <* 0.05, ** indicates *p <* 0.01, *** indicates *p <* 0.001.

RhoA is known to activate cell migration by re–organizing the actin cytoskeleton [29]; therefore, we analyzed the distribution of F–actin under NPFFR2 overexpression by staining with Phalloidin. The fluorescence intensity of phalloidin increased by NPFFR2 overexpression in Huh7 cells (Figure 6A) and decreased by NPFFR2 depletion in SNU475 cells (Figure 6B).

Next, we investigated the function of NPFFR2 in YAP activation, as YAP signaling is a downstream pathway of Rho signaling, and the Hippo pathway is regulated by GPCR signaling [30]. Cellular mechanical stress, including actin remodeling, is known to regulate YAP activity. This is because LATS—a protein that inhibits YAP activity by phosphorylating it, inhibiting its entry into the nucleus, and causing it to degrade in the cytoplasm—is activated when F–actin is disrupted [31]. We analyzed whether NPFFR2 regulates YAP activity. Immunofluorescence staining using YAP antibody revealed that the fluorescence intensity and nuclear localization of YAP increased with the transient overexpression of NPFFR2 in Huh7 cells (Figure 6A) and decreased by depletion of NPFFR2 in SNU475 cells (Figure 6B).

The regulation of YAP by NPFFR2 was also confirmed by analyzing downstream genes—*CTGF* and *Cyr61*—actively transcribed by YAP. Transcription was analyzed by real–time PCR in cells where NPFFR2 was overexpressed or depleted. NPFFR2 overexpression increased the transcription of *CTGF* and *Cyr61* in Huh7 and SNU449 cells (Figure 6C), whereas NPFFR2 depletion decreased it in SNU739 and SNU475 cells (Figure 6D). Since YAP is well known to induce anchorage-independent cell growth [32], we finally validated whether NPFFR2 controls this tumorigenic function using the soft agar assay. Anchorage–independent cell growth was dramatically increased in Huh7 cells stably overexpressing NPFFR2. This implies that NPFFR2 may function on tumorigenesis. Taken together, these data suggest that the malignant function of NPFFR2 is acquired from activating RhoA/F–actin/YAP signaling.

## 4. Discussion

GPCR signaling cascades play a central role in cancer progression, including growth, metastasis, angiogenesis, stemness, and immune response. As GPCRs are the largest protein family, identifying dysregulated members among numerous GPCRs, along with cancer type, is critical for the clinical utility of the GPCR pathway in cancer. Here, we report that NPFFR2, a neuropeptide GPCR, overexpressed in HCC, is an indicator of poor prognosis of HCC, and its expression is increased in more advanced stages. Depletion of NPFFR2 expression efficiently suppressed the survival, migration, and invasion of various cancer cells, and overexpression of NPFFR2 promoted migration, invasion, and anchorage-independent cell growth. Thus, for the first time, we report that NPFFR2 acts in cancer malignancy. Considering its expression and function, we suggest that targeting of NPFFR2 may be a potential therapeutic option for advanced HCCs.

Activated GPCRs trigger signaling cascades by binding to heterotrimeric G proteins. Moreover, NPFFR2 may mediate more complicated cellular responses because it couples with multiple Gα subunits. We focused on the RhoA mechanism since this protein is commonly activated by multiple Gα proteins and promotes cell migration and invasiveness, and identified that the function of NPFFR2 on tumor progression depends on the RhoA pathway. The ROCK inhibitor completely inhibited the effect of NPFFR2 on cell migration and invasion, and overexpression and depletion studies revealed that NPFFR2 positively controls the ROCK/F–actin/YAP signaling axis. Moreover, since YAP signaling is activated in HCC to promote malignant progression [33,34], the YAP/TAZ pathway has emerged as an attractive target for HCC treatment [35]. This adds to the advantage of selecting NPFFR2 as a therapeutic target.

NPFF, the peptide ligand that activates NPFFR2, also showed a similar phenotype to NPFFR2 overexpression; however, the effect was much lower, and the activated signaling was different. There are other GPCRs activated by NPFF, such as NPFFR1 and MAS [36], and NPFFR2 can also be activated by other ligands, such as NPVF, PrRP, and kisspeptin [37]. Since NPFF and NPFFR2 are not mutually exclusive, there is a difference in the degree of their effect on cancer cells and in signal transduction. Therefore, it is critical to develop antagonists and inhibitors that specifically control NPFFR2.

This is the first study to demonstrate the close association of NPFFR2 to cancer progression. As NPFFR2 has an intrinsic function in the nervous system, further studies are needed to determine the effect of its inhibition on individuals. However, NPFFR2 expression in HCC patient tissues and HCC cell lines was higher than that of normal liver tissue or normal cells, respectively, and in cells where NPFFR2 was overexpressed, siRNA tended to deplete NPFFR2 more effectively, resulting in cytotoxicity. Therefore, we expect NPFFR2 inhibition to have an effective cytotoxicity in cancer cells compared to normal tissues. Fewer side effects are also expected because NPFFR2 knockout mice do not exhibit problems other than psychological differences [38]. Abnormal overexpression of NPFFR2 in some cancers, as shown in HCC tissues and cell lines, is advantageous for an anticancer target. In particular, the discovery of novel factors contributing to malignancy with increased expression in advanced HCC is more valuable because liver cancer is difficult to detect at an early stage. In addition, since NPFFR2 overexpression was confirmed in cancer cell lines derived from other organs, it is expected to be an effective anticancer target in other cancer types. 

## 5. Conclusions

In this study, we identified a novel effect of NPFFR2 on HCC progression. The expression of NPFFR2 in HCC patients was related to poor prognosis and was particularly increased in advanced HCCs. Our in vitro results showed that inhibition of NPFFR2 was effective in preventing cancer progression through inhibition of cancer survival, migration, and invasion. Here, NPFFR2 represents a potential anticancer target among GPCRs, and its value would be further verified through follow-up studies.

## Data Availability

Data generated from TCGA-LIHC dataset in present study were downloaded from KM plotter (http://www.kmplot.com) and UALCAN database (http://ualcan.path.uab.edu).

## Supplementary Materials

The following supporting information can be downloaded at: https://www.mdpi.com/article/10.3390/cancers14235850/s1, Figure S1: mRNA expression of NPFFR2 in cancer cell lines from various origins was analyzed by RT-PCR, Figure S2: (A) Invasion assay was performed in Huh7, SNU475, and SNU739 cell lines in which NPFFR2 was stably overexpressed. (B) Graphs were obtained from three independent experiments, and each experiment was performed in triplicate. (C) Overexpression of NPFFR2 was analyzed using real-time PCR. The expression was calculated from the mean CT value of technical triplicates and normalized to B2M. Full pictures of the Western blots are presented in Supplementary Materials.
