# Supplementary material for: NPFFR2 Contributes to the Malignancy of Hepatocellular Carcinoma Development by Activating RhoA/YAP Signaling
Source: Cancers (Basel). 2022 Nov 27;14(23):5850. doi: 10.3390/cancers14235850 (PMC9737590; doi:10.3390/cancers14235850)
Supplement: Supplementary file 1 [file cancers-14-05850-s001.zip › cancers-2013677-supplementary.pdf]

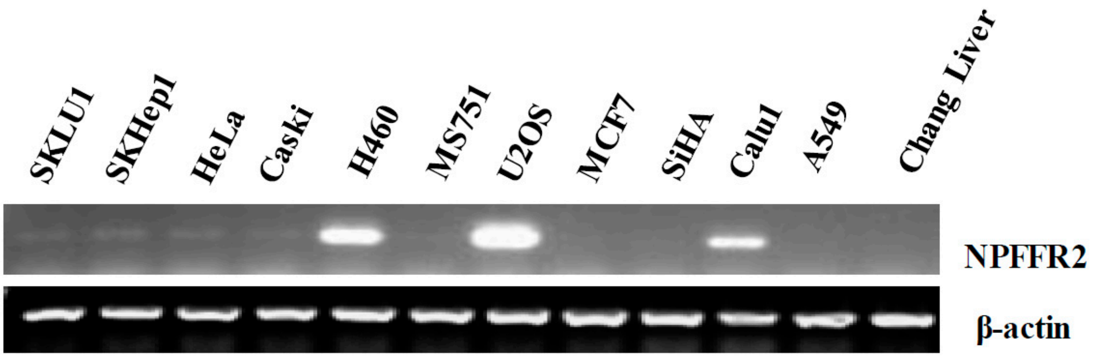

Figure S1. mRNA expression of NPFFR2 in cancer cell lines from various origins was analyzed by RT-PCR.

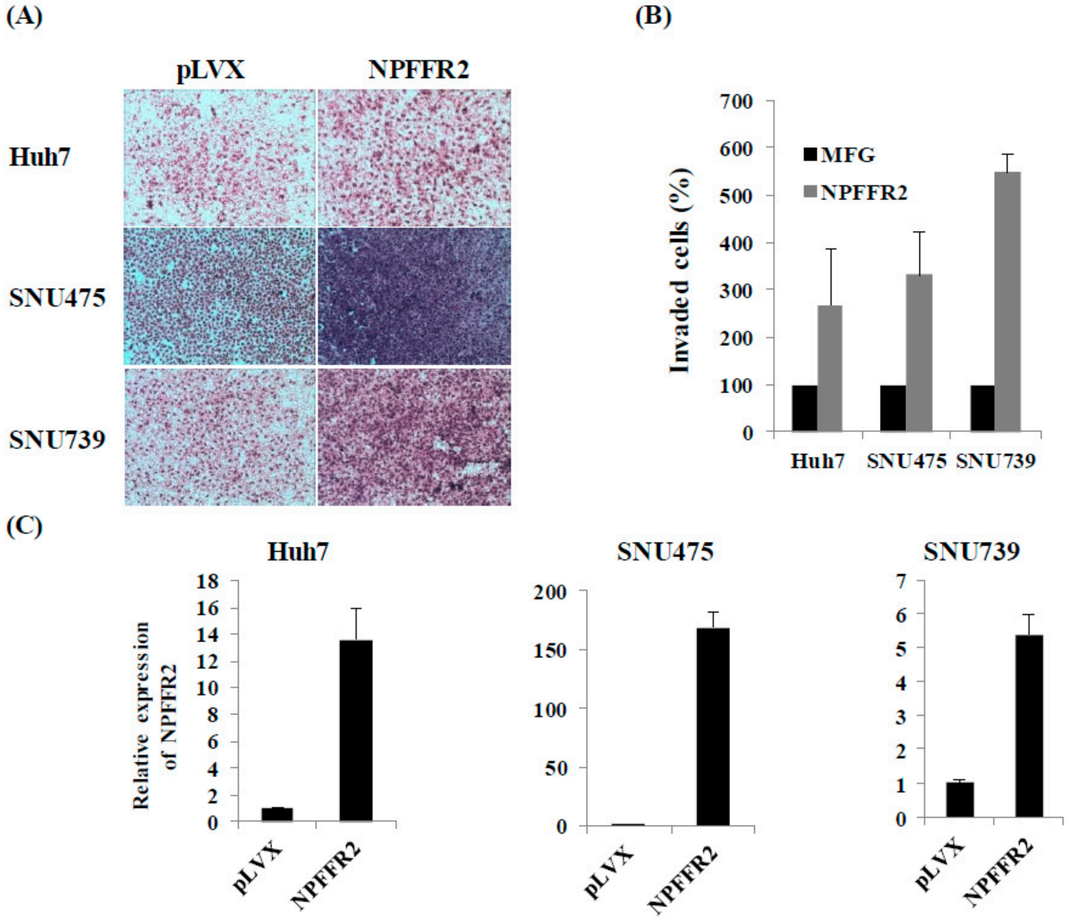

Figure S2. (A) Invasion assay was performed in Huh7, SNU475, and SNU739 cell lines in which NPFFR2 was stably overexpressed. (B) Graphs were obtained from three independent experiments, and each experiment was performed in triplicate. (C) Overexpression of NPFFR2 was analyzed using real-time PCR. The expression was calculated from the mean CT value of technical triplicates and normalized to B2M.
